# The predictive power of stock market’s expectations volatility: A financial synchronization phenomenon

**DOI:** 10.1371/journal.pone.0250846

**Published:** 2021-05-20

**Authors:** Nicolás Magner, Jaime F. Lavin, Mauricio Valle, Nicolás Hardy

**Affiliations:** 1 Facultad de Economía y Negocios, Universidad Finis Terrae, Santiago, Chile; 2 Escuela de Negocios, Universidad Adolfo Ibáñez, Santiago, Chile; University of Almeria, SPAIN

## Abstract

We explore the use of implied volatility indices as a tool for estimate changes in the synchronization of stock markets. Specifically, we assess the implied stock market’s volatility indices’ predictive power on synchronizing global equity indices returns. We built the correlation network of 26 stock indices and implemented in-sample and out-of-sample tests to evaluate the predictive power of VIX, VSTOXX, and VXJ implied volatility indices. To measure markets’ synchronization, we use the Minimum Spanning Tree length and the length of the Planar Maximally Filtered Graph. Our results indicate a high predictive power of all the volatility indices, both individually and together, though the VIX predominates over the evaluated options. We find that an increase in the markets’ volatility expectations, captured by the implied volatility indices, is a good Granger predictor of an increase in the synchronization of returns in the following month. Estimating, monitoring, and predicting returns’ synchronization is essential for investment decision-making, especially for diversification strategies and regulating financial systems.

## 1. Introduction

In recent years, interest in implementing strategies of international diversification, motivate investors to search for assets, markets, and regions that protect them against economic and financial shocks. But cross-market and cross-asset linkages, the interconnectedness of financial markets, stock returns co-movement [1,2], and specifically, episodes of higher synchronization of returns are key elements that jeopardize the effectiveness of such strategies. This behavior is part of the financial system, as we witnessed during the subprime crisis when markets react with heightened returns synchronization. This phenomenon demonstrates the necessity to look at the financial market as a complex system.

Financial markets are a typical example of a complex system. Characterized by numerous entities and interaction rules that lead to collective behaviors that generally depend on the interactions between the entities belonging to the system. A complex financial system has multiple assets and markets, where investors value financial assets according to their expectations, market conditions and consequently, make investment decisions generating synchronization between the prices and returns of assets and markets [3]. This behavior is a recurrent phenomenon due to the growing economic and financial interconnectedness of countries and markets. Evidence shows that greater global economic and political openness fosters integration and interconnection at the capital markets level, transforming them into larger and more complex financial systems. As financial shocks show, a highly interconnected financial system is prone to suffer rare events such as the Subprime crisis and the Covid-19 pandemic, where local shocks were amplified, spread, and quickly turned into a global turmoil.

Useful network methods for study markets´ behavior are the Minimum Spanning Tree (MST) and the Planar Maximally Filtered Graph (PMFG). With these techniques, it is possible to build a connected network of financial assets to identify topological features related to the emergence of returns synchronization in stock markets [4]. For example, evidence indicates that during synchronization of returns or collective behavior–where financial assets exhibit a similar tendency, the asset´s network displays a change in their topology related to the “small–world” property of Watts and Strogatz [5]. Under such events, this phenomenon facilitates a more efficient coupling among the network’s components and increases return synchronization [6].

Additionally, several studies demonstrate the usefulness of these measures for making investment decisions. Guo et. al., [7] use the MST to categorize the Chinese stock market in central and peripheral stocks, finding that the network’s peripheral ones, being less synchronized with the rest of the market stocks, offer a similar return but with lower levels of risk, making them more attractive to increase portfolio diversification. From a systemic perspective, Magner et al. [4] use the length of the MST (MSTL) and the correlation network, to represent the temporal dynamics of the synchronization phenomenon of regional stock markets of America, Europa, Asia, and Oceania, and study how this dynamic has predictive power on the realized volatility of the stock indices of the main exchanges of the world. Their results provide practical implications for the investment management industry and for the regulator´s viewpoint.

Currently, an important gap in the literature relates to the understanding of the factors of the synchronization of returns in financial markets. This phenomenon is important because as the evidence indicates, an increase in synchronization leads to a rise in the systemic risk of the markets and a decrease in the effectiveness of diversification as a risk management tool [8]. As the literature points out, both variables are fundamental elements to monitor the stability of financial markets and adequately manage the risk of investment portfolios.

A conjecture of the stock market synchronization links to the execution of investment strategies. When facing an unexpected change in the expected volatility due to variations in uncertainty, investors will execute similar asset reallocation decisions. For instance, investors implementing the typical "flight to quality" strategy, whereby they sell (buy) stocks and buy (sell) bonds, generating a greater synchronization of asset returns in the short term. Similarly, a shock caused by a war or a pandemic such as Covid-19 will increase future uncertainty causing investors to rebalance their portfolios towards safe-haven assets. Consequently, this collective behavior causes similar decisions in the agents that enhance the synchronization of financial assets in the entire market.

Ample evidence relates volatility, as a simple measure of risk and uncertainty, as one of the main measurements used to describe and quantify financial asset return fluctuations. For example [9–12], show a negative, contemporaneous, and asymmetric relationship between changes in volatility indices and stock indices’ returns. However, despite this robust body of empirical evidence of the literature, we still know little about the influence of volatility on the synchronization of returns. Nowadays, this issue is still a critical phenomenon for managing investment portfolios and monitoring financial stability and systemic risk [13–15].

Shocks can generate volatility spillovers. A phenomenon related to systemic risk, which can arise through interlinkages between the financial system components so that individual failure or malfunction has repercussions around the financial system. However, little research links international volatility spillovers to global financial systemic risk. In this sense, given the increasing size and sophistication of derivatives markets, volatility spillovers can now be studied with stock implied volatility as an ex-ante risk-neutral expectation of future volatility and directly available daily or even intraday frequency. Relatively few studies have explored implied volatility spillovers across countries and asset classes. This paper is one of these studies.

The purpose of this paper is to evaluate the predictive power of the stock market’s implied volatility indices on the synchronization of stock market returns. For this, we represent the volatility expectations of the markets using the implicit volatility indices’ behavior and we estimate markets’ returns synchronization applying asset trees methodologies. Specifically, to capture the changes in uncertainty levels in the financial markets, we use the VIX and two alternative indices, the European VSTOXX and the Asian VXJ. These indices have historically been the object of research due to their demonstrated capacity to represent the fear of financial markets and for its effects on financial market’s investment decisions, coverage, and regulatory aspects.

We use the length of the Minimum Spanning Tree (MSTL) and the length of the Planar Maximally Filtered Graph (PMFGL) for measuring the synchronization of asset returns [16,17]. As the literature states an increase (decrease) in these parameters indicates a lower (higher) correlation within the asset network, and consequently, a lower (higher) synchronization of returns. To cover the most considerable portion of the leading world stock markets, we include 26 stock indices of markets made up of North America, Latin America, Europe, Asia, and Oceania. Then, to assess the relationship between the stock market’s volatility expectations and the synchronization of returns, we run tests to estimate Granger causality. In this sense, we carry out tests within the sample using statistical series between 2001 and 2020 with monthly frequency. Finally, to dig deeper into the predictive power of volatility indices, we applied several out-of-sample tests with different sizes of estimation windows.

Applying these methodologies, this paper contributes to the literature on volatility spillover effects in equity markets, attempting to determine the extent to which financial globalization and increased regional integration affect interdependence among equity markets. There is broad empirical literature investigating the effects of further financial integration on economic growth and investment. Further integration increases local returns’ sensitivities to common world (regional) shocks and higher cross-market equity synchronization. As a consequence, the potential of country, regional and global diversification strategies may decrease. In an attempt to place ourselves on the other side of the current literature, we use three implied volatility stock indices to forecasting cross-market equity synchronization, adding a novel point of view of the usefulness of implied volatility indices.

Our main results indicate a significant predictive power of all the implicit volatility indices at the global and regional networks of stocks. Also, we find a high predictive power of the VIX, finding a negative relationship between all the volatility indices and the stock markets synchronization levels, represented by changes in the MSTL and the PMFGL. In other words, the evidence shows that by increasing the expected volatility captured by rises in the variations of the VIX, VSTOXX, and VXJ, a significant signal of future increment in the global and regional synchronization of the equity markets is generated by decreasing the lengths of the MST and PMFG. In addition to these results, we apply a Structural VAR that suggests the existence of Granger-causality. This predictability seems to go from the VIX, VSTOXX, and VXJ to the MSTL, providing strong evidence that the implicit volatility of the stock market generates future stock market synchronization.

Our evidence has important implications for investors, fund managers, and market regulators. First, our work shows that an increase in the implicit market volatility is the forerunner of a future increment in the synchronization of the returns of the stock markets, which would imply a greater level in the systemic risk and a decrease in the benefits of portfolio diversification as a risk minimization tool. In this sense, from an investor’s point of view, our research helps them monitor one of the factors associated with the synchronization of equity market returns. Second, portfolio managers can use these results to estimate return timing thresholds that would allow them to anticipate high synchronization events and their consequent effects on the effectiveness of portfolio diversification. Finally, from the viewpoint of regulators, our paper highlights the role of implicit volatility indicators to explain future events of high financial synchronization. This issue present in high turmoil and high uncertainty episodes significantly increases systemic risk levels in financial markets [18].

A word of caution. This research does not study the structural links between implied volatility and stock market synchronization. For this proposal, we need a structural model. We only evaluate the predictive ability of three implied volatility indices via Granger-causality and forecasting regressions over the stock market synchronization, which are useful to assess whether a variable has the predictive ability, not whether its “cause” other variables to change. In this regard, our work is the first step for studying the possible links between the implied volatility and uncertainty in financial markets and its predictive effects on asset networks.

The rest of the paper is organized as follows. In section 2, we explain in detail the forecasting methodology and models. In section 3, we present and discuss the results. In section 4, we conclude.

## 2. VIX and stock markets behavior

There is evidence of an interrelation between implied market volatility, contemporaneous and future stock returns, and economic uncertainty. Known as the investor fear gauge, since high levels of the Chicago Board Options Exchange Volatility Index (VIX) coincide with high degrees of market turmoil. VIX measures market expectations of stock return volatility and corresponds to a measure of the ex-ante risk-neutral expectation of future volatility of American stocks [9,19–21]. Calculated initially from S&P100 stock index options, from 2003, the VIX is estimated from the S&P500 index option prices. Evidence indicates that VIX predicts returns on stock market indices, suggesting that implied volatility is a risk factor for security returns. Nowadays, it is the best gauge to forecast volatility of equities, and it is an indicator highly used by investors as a measure of stock market uncertainty. For instance, Banerjee et. al. [22] state that VIX has a robust predictive capacity for future stock returns evidencing a positive relationship between S&P500 future performance and VIX evolution.

As a tool to gauge market volatility, some traders use VIX as a stock market timing tool. Based on the observation that high levels of VIX often coincide with market bottoms, VIX seems to indicate "oversold" markets. Therefore, traders can take long positions in the market in anticipation of an increase after VIX is high. Giot [23] tests if high levels of VIX indicate oversold stock markets by dividing the VIX price history into equally spaced rolling percentiles and examining the returns on the S&P100 for various future holding periods up to 60 days for each of these percentiles. He finds that for very high (low) levels of VIX, future returns are always positive (negative) and that negative (positive) contemporaneous returns are associated with increased (decreased) implied volatility. These findings suggest that too high levels of VIX may signal attractive buying opportunities.

In the same vein, Copeland and Copeland [24] find that changes in VIX are statistically significant leading indicators of daily future market returns and a tool for improving a stock portfolio’s yield. They state that on days that follow increases in VIX, portfolios of large-capitalization stocks outperform portfolios of small-capitalization stocks and value-based portfolios outperform growth-based portfolios. Similarly, on days following a decrease in VIX, the opposite happens with the latter portfolios’ performance. The implication they state is that market timing using VIX may be appropriate for portfolio yield strengthening.

Finally, VIX also has implications for economic uncertainty. Exploring the dynamic co-movements between macroeconomic policy uncertainty, stock market returns, and stock market implied volatility, Antonakakis et. al [25] find dynamic correlations between macroeconomic policy uncertainty and stock market returns are mainly negative. Also, an increase in the stock market implied volatility–measured by VIX- coupled with a higher macroeconomic policy uncertainty diminish stock market returns while it increases economic policy uncertainty.

VIX fluctuations not only impacts markets return and economic uncertainty in local terms, but also has a leading role in the context of the international markets. Investigating the cross-market relations of volatility indexes with US and non-US stock market returns, Shu et. al [26] report a pervasive VIX influence at both US and non-US stock markets. They find that information flow is unidirectional from VIX to the stock market, being the VIX change a critical determinant of stock market returns. They also indicate that as VIX plays a role in the spillovers’ direction, investors can use it to predict stock market movement both in the US and the international markets.

Finally, when comparing the VIX versus other volatility indexes such as VSTOXX and VKOSPI, Shu et. al [26] show that VIX is the most significant contributor of spillovers towards other volatility indexes, pointing VIX with a leading role in the international markets. Similar results document Kang et. al [27] when analyzing the dynamic pattern of spillover and connectedness between a broad set of financial assets, find that there are spillovers between VIX and VSTOXX and that the latter volatility index acts as a net transmitter of shocks, especially during periods of turmoil in European financial markets.

## 3. Materials and methods

### 3.1 Data

We utilize daily data provided by Bloomberg and Refinitiv from July 2001 to July 2020, totaling 223 months. As independent variables, we use three stock market implied volatility indices: CBOE VIX index (VIX), EURO STOXX 50 Volatility (VSTOXX), and volatility Index Japan (VXJ). With these indices, we carry out tests to predict the MSTL monthly variation for four regional markets: North America, Latin America, Europe, Asia, and Oceania. To compute the MSTL we take 26 stock market indexes (see Table 1 for details).

**Table 1:** Stock country indices to estimate MSTL’s region.

MSTL Region	Indices
North America	S&P500, NASDAQ from USA and TSX from Canada.
Latin America	IPC from Mexico, BOVESPA from Brazil, IPSA from Chile, MERVAL from Argentina, IGBVL from Peru.
Europe	FTSE from UK, CAC from France, DAX from Germany, IBEX from Spain, MIB from Italy, AEX from Holland, OMX from Sweden, RTS from Russia, and SMI from Swiss.
Asia	NIKKEI from Japan, HANG-SENG from Hong Kong, KOSPI from Korea, TSE from Taiwan, JSE from Indonesia, KLCI from Malaysia, and ST from Singapur.
Oceania	ASX from Australia and NZSE from New Zealand.

This table indicates the stock indices considered to calculate the MSTL for each region. Global MSTL is calculated with all stock indices. Source: Authors’ elaboration.

**Fig 1:**
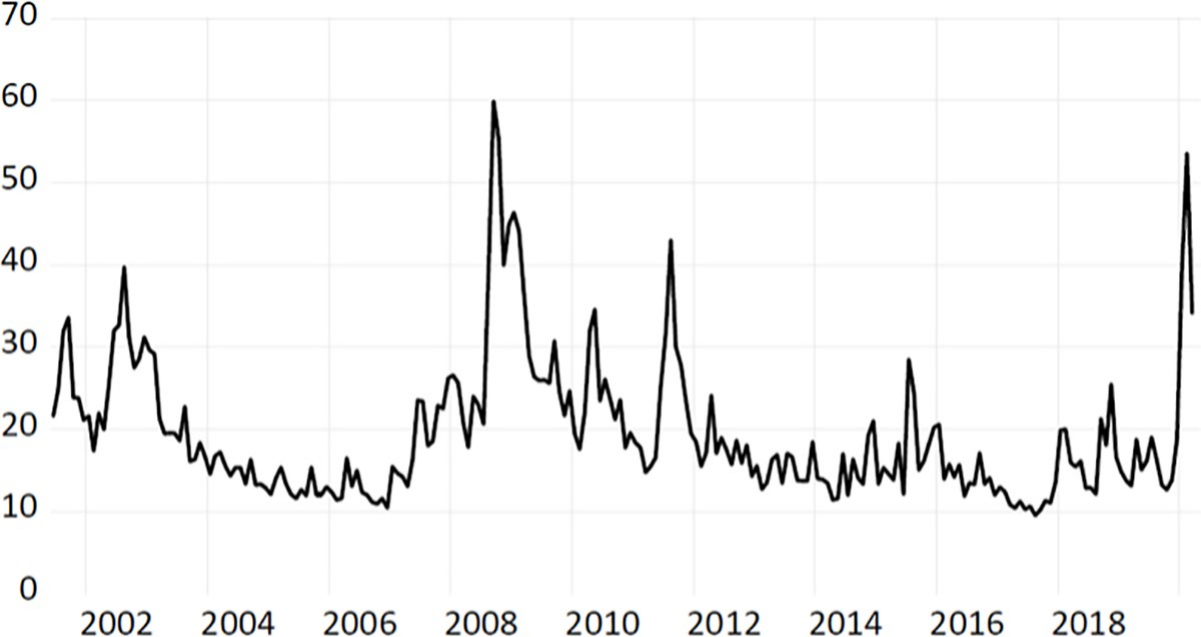
VIX levels and time evolution. Note: This figure depicts the time series evolution of the VIX between the years 2002–2019.

Table 2 exhibits our summary statistics for the three series of implied volatility at the monthly frequency. The series is considered here both in levels (Panel A) and first log-differences (Panel B). Some features are worth mentioning. First, the maximum values for our measures of implied volatility coincide with the 2009 global financial crisis (notice the spike in Fig 1). Second, we study the existence of unit-roots in our series through a Phillips-Perron test; as reported by a vast literature (e.g., Yang and Zhou [19]), the implied volatility series (Panel A) does not seem to be stationary.

**Table 2:** Summary statistics of monthly implied volatility indices.

	Panel A			Panel B		
	VIX	VSTOXX	VXJ	DLOG(VIX)	DLOG(VSTOXX)	DLOG(VXJ)
Mean	19.461	23.509	24.443	0.002	0.002	0.000
Median	16.800	21.085	22.730	-0.016	-0.028	-0.020
Maximum	59.890	61.340	96.690	0.853	0.901	0.908
Minimum	9.510	11.986	12.030	-0.486	-0.516	-0.641
Std. Dev.	8.467	9.443	9.462	0.208	0.199	0.212
Skewness	1.913	1.482	3.013	0.633	0.602	0.765
Kurtosis	7.523	5.191	19.323	4.493	4.609	5.649
Jarque-Bera stat	330.506	127.987	2850.790	35.921	37.870	87.711
p-value	0.000	0.000	0.000	0.000	0.000	0.000
Phillips-Perron stat	-0.888	-0.941	-1.433	-20.109***	-20.271***	-22.835***
p-value	0.33	0.308	0.142	0.000	0.000	0.000
Observations	226	226	226	225	225	225

This table reports the summary statistic of monthly implied volatility indices from July 2001 to July 2020. Panel A shows implied volatility indices’ levels; Panel B shows implied volatility indices on the first difference. Source: Authors’ elaboration.

**Fig 2:**
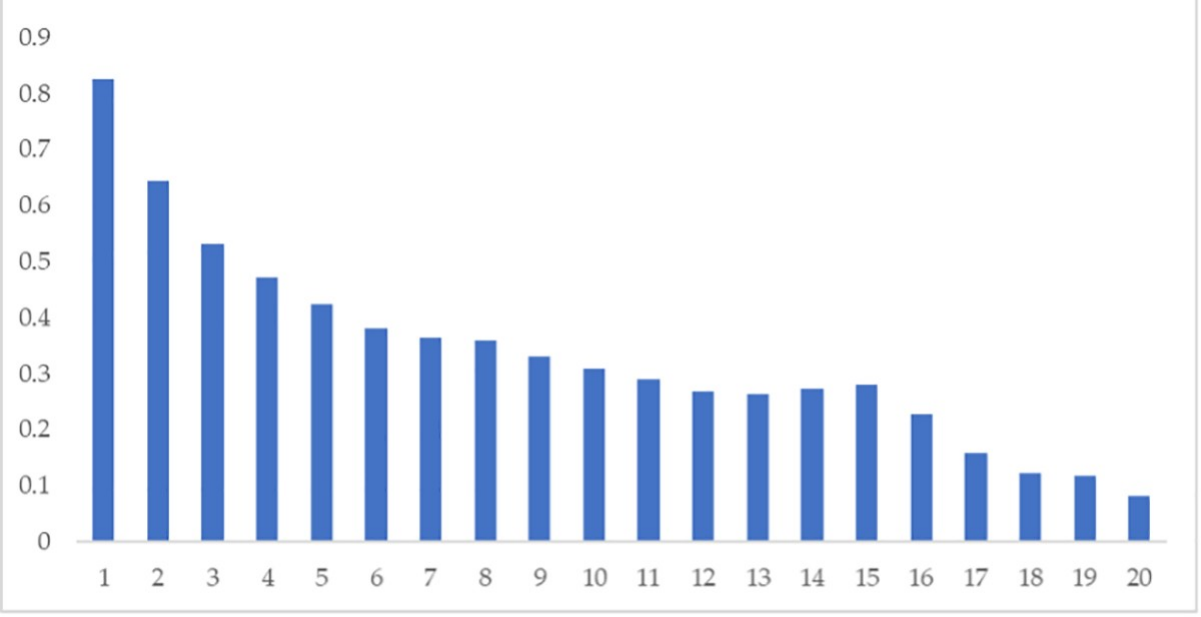
VIX autocorrelation graph. Note: This figure depicts the autocorrelation graph for VIX levels with lags from 1 to 20.

Moreover, Fig 2 shows that the autocorrelations are strong, and tend to decay linearly rather than exponentially, a common feature of the unit-root series. In contrast, our series in Table 2 Panel B strongly reject the null hypothesis of unit-roots in all cases. For this reason, we consider the first log-differences (Table 2 Panel B) in all our econometric specifications. Third, a note of caution: for completeness and illustrative purposes, we report sample moments for both Table 2 Panel A and Panel B series. However, we acknowledge that our series in Table 2 Panel A are not stationary neither ergodic. Hence the existence of population moments (or the convergence of sample moments to population moments) may be highly debatable. In this sense, for Table 2 Panel A, our Means, Std. Dev, Skewness, and Kurtosis may be somewhat misleading. Finally, both Panels of series tend to be fat-tailed and, to some extent, skewed (at least for Panel A); not surprisingly, according to the Jarque-Bera test, the null of Normality is strongly rejected in every case (especially for Table 2 Panel A).

### 3.2 Networks analysis

A simple transformation of the matrix of linear correlation between return assets into an equivalent distance produces a connected network studied in numerous works. In this network, the nodes correspond to the assets, and the edges that join them represent the distances obtained from those correlations. All the nodes are connected with them, so if the network has *N* nodes, there will be *N(N—1)/2* edges.

We consider that there are *N* assets priced *P*_*i*,*t*_ for the asset *i* at time *t*. The logarithmic return of the asset *i* given by *r*_*i*,*t*_ *=* ln*P*_*i*,*t*_*−*ln*P*_*i*,*t-1*_ is computed. In our case, the returns are daily. The synchronization between the assets i and j is captured with the simple linear correlation defined as

$$\rho_{ij}=\frac{\left\langle r_{i}r_{j}\rangle -\langle r_{i}\rangle \langle r_{j}\right\rangle }{\left[\langle r_{i}^{2}\rangle -\langle r_{i}\rangle^{2}][\langle r_{j}^{2}\rangle -\langle r_{j}\rangle^{2}\right]}$$ [Eq 1]

where *⟨*…*⟩* indicates the average over a period, which for our case, corresponds to a month. The vector ***r***_*i*_ corresponds to the return vector of the asset *i*. The same for the asset *j* with the vector ***r***_*j*_. The resulting correlation matrix contains the correlations between each pair of assets, which are transformed to a distance metric, such that *d*_*ij*_ = (2(1−*ρ*_*ij*_))^1/2^, represents the distance between assets *i* and *j*. Thus, a correlation *ρ*_*ij*_ = −1 indicates a maximum distance of *d*_*ij*_ = 2, while *ρ*_*ij*_ = 1 indicates a minimum distance of *d*_*ij*_ = 0 [2]. The asset distance matrix is part of the input to find a minimum distance asset tree.

To filter the information contained in this completely connected network, it is possible to find subsets of the network or find asset trees. Thus, it is not necessary to have all possible connections between nodes. A Minimum Spanning Tree (MST) links all the nodes of the network without cycles so that the distance path made when passing through all the nodes is minimal. The construction of this asset tree is very well documented (see, for example, [16] and [28]), and we briefly describe it in the methodology.

In this case, the corresponding MST is a simplified version of the complete asset network with *(N-1)* edges. The distance traveled to pass through each node we call the length of the MST (MSTL). Obviously, for each month *t*, the MST will be different, because the correlations between assets are dynamic, and therefore there will be variation in the length of the MST. The MSTL is

$$L(t)=\frac{1}{N-1}\sum_{d_{ij}^{t}\in T^{t}}d_{ij}^{t}$$ [Eq 2]

where *T*^*t*^ represents the asset tree in month *t*. The sum of the distances is done on all the edges of *T*^*t*^. The length is divided by *N-1*, the number of edges of the tree to obtain a standardized measure of the length. The MSTL is a representation of the level of synchronization of the assets. The variation of the MSTL is named *VMSTL*_*t*_ *=* ln*(MSTL*_*t*_*)—*ln*(MSTL*_*t-1*_*)*, whether negative (positive), indicates a contraction (expansion) of the tree, i.e. an increase (decrease) in synchronization of returns.

Another alternative for simplifying the entire network of assets is the Planar Maximally Filtered Graph (PMFG) (see Tumminello et. al [29] and Tumminello et. al [30] for details of the methodology). In this case, the graph is a subset of the entire network, but with *3N-6* edges, i.e., it has more information than the MST. The resulting PMFG network contains the MST [28]. For this network, the length of the PMFG (PMFGL) is determined in the same way as in Eq 2; the summation is done on the PMFG network and not on the MST. Obviously, for the same period, the PMFGL will always be longer than the MSTL because the former admits a greater number of edges.

### 3.3 Forecasting model and evaluation

We build two forecasting models to evaluate the predictive power of the VIX, VSTOXX, and VXJ. In Table 3, Panel A, we name "core models" to forecasting models for our in-sample and out-of-sample tests, that include the variation of the VIX (henceforth, VVIX), the variation of the VSTOXX (henceforth, VVSTOXX), the variation of the VXJ (henceforth, VVXJ), and include a principal component that combines the three previous implied volatility indices. Second, for our out-of-sample tests, we named "benchmark models," a model *AR(p)* used in the forecasting literature to compare predictive power [31,32]. All models are estimated using heterogeneous autoregressive (HAR) methodologies (See Table 3).

**Table 3:** Forecasting models.

	Panel A–Core Models
(1)	${VMSTL}_{i,t}=c+\beta_{i}*{VVIX}_{t-1}+\gamma_{i,k}*{VMSTL}_{i,t-k}+e_{i,t}$
(2)	${VMSTL}_{i,t}=c+\beta_{i}*{VVSTOXX}_{t-1}+\gamma_{i,k}*{VMSTL}_{i,t-k}+e_{i,t}$
(3)	${VMSTL}_{i,t}=c+\beta_{i}*{VVXJ}_{t-1}+\gamma_{i,k}*{VMSTL}_{i,t-k}+e_{i,t}$
(4)	${VMSTL}_{i,t}=c+\beta_{i}*{VVIX}_{t-1}+\beta_{i}*{VVSTOXX}_{t-1}+\beta_{i}*{VVXJ}_{t-1}+\gamma_{i,k}*{VMSTL}_{i,t-k}+e_{i,t}$
(5)	${VMSTL}_{i,t}=c+\beta_{i}*{PC}_{t-1}+\gamma_{i,k}*{VMSTL}_{i,t-k}+e_{i,t}$
	Panel B–Benchmark Models
(2)	${VMSTL}_{i,t}=c+\gamma_{i,k}*{VMSTL}_{i,t-k}+e_{i,t}$

Source: Authors’ elaboration.

*VMSTL*_*t*_ is the variation of the global minimum spanning tree length in the month *t*, *VVIX*_*t-1*_ is the first lag of the variation of VIX in the month *t*, *VVSTOXX*_*t-1*_ is the first lag of the variation of VSTOXX in the month *t*, *VVXJ*_*t-1*_ is the first lag of the variation of VXJ in the month *t*, *PC*_*t-1*_ is the first lag of the principal component estimate with the varimax rotated method that combine the VIX, VSTOXX, and VXJ in the month *t*, *VMSTL*_*t-k*_ is the global minimal spanning tree length in *k* lags, and *e*_*t*_ is disturbance error in the month *t*.

We use *AR(p)* models as benchmarks due to the autocorrelation and stationarity of the MSTL time series. We perform autocorrelation and stationarity analysis of the MSTL time series, finding persistence and stationarity, which allows ruling out benchmark models of random walks.

As mentioned, this paper aims to test the existence of Granger causality from the implied volatilities indices toward the synchronization of returns of the assets network. In this sense, we consider the following null hypothesis *H*_0_: *β*_*i*_ = 0. This null hypothesis posits that the implied volatility indices have no role in predicting the variation of the asset structure. We test these hypotheses both in-sample and out-of-sample focusing on one-step-ahead forecasts following Clark and McCracken [33].

We evaluate the in-sample test using the t-statistic associated with the coefficient of the minimal spanning tree length (MSTL). The Central Limit Theorem for weakly stationary processes (e.g., Hamilton and Susmel [34] requires a proper estimation of the long-run variance. For this purpose, we use HAC standard errors following Newey and West [35,36] because the VMSTL time series is autoregressive and has seasonal factors.

To mitigate the usual overfitting problems associated with in-sample analyses, we also consider out-of-sample analyses. For this, we use the ENCNEW test proposed by Clark and McCracken [33]. This test is important since our benchmark models are nested in our core models (See Table 3); hence, the usual tests of forecast evaluation become degenerate under the null hypothesis [37–39]. The ENCNEW has a non-standard asymptotic distribution, but critical values for one-step- ahead of forecasts are tabulated in Clark and McCracken [33]. In particular, under the null hypothesis, the asymptotic distribution of the ENCNEW is a function of Brownian motions depending on the number of the excess parameters in the nesting model (in our case, this parameter is 1 or 3, depending on the model), on the scheme being used to estimate our models (in our case, expanding windows), and on the parameter defined as the limit of the ratio *P/R*, where *P* is the number of one-step-ahead forecasts and *R* is the size of the first expanding window used in the out-of-sample analysis. See West [39] and Clark and McCracken [33] for a survey on out-of-sample evaluation.

In this way, on the one hand, we estimate our models with all the available observations for our in-sample analyses. On the other, for our out-of-sample analyses, we split the sample in two: an initial estimation window of size *P* and an evaluation window of size *R*, such that *T = P + R*, where *T* is the total number of observations. To avoid any concern about our data’s specific splitting, we use three different approaches to split our sample. First, we use one-third of our observations for initial estimation and two-thirds for evaluation (this means *P/R = 2*). Second, we use a half of our observations for initial estimation and the other half for evaluation (this means *P/R = 1*). Third, we use two-thirds of our observations for initial estimation and one-third for evaluation (this means *P/R = 0*.*4*).

### 3.4. Impulse response function and forecasting error variance decomposition

In this stage of the analysis, we follow Hamilton [40] notation and results for VAR(p) processes. Let *Y*_*t*_ be a (nx1) vector containing the values of n variables at time t. Suppose that the dynamics are determined by a *p*th-order VAR as follows:

$$Y_{t}=c+\rho_{1}Y_{t-1}+\cdots +\rho_{p}Y_{t-p}+\varepsilon_{t}$$ [Eq 3]

Where *c* is an (nx1) vector collecting the drifts in each equation, *ρ*_*i*_ are (nxn) coefficients matrices and *ε*_*t*_ is i.i.d N(0, Ω).

According to Wold (or MA(∞)) representation theorem, every weak-stationary and purely non-deterministic process allows the following representation:

$$Y_{t}=c+\varepsilon_{t}+\Psi_{1}\varepsilon_{t-1}+\Psi_{2}\varepsilon_{t-2}+\Psi_{3}\varepsilon_{t-3}+\cdots$$ [Eq 4]

In this sense, the matrix Ψ_s_ has the following interpretation

$$\frac{\partial Y_{t+s}}{\partial \varepsilon_{t}\prime }=\Psi_{s}$$ [Eq 5]

Notice that a sequence of row i, column j element of Ψ_s_ (say $\frac{\partial Y_{i,t+s}}{\partial \varepsilon_{jt}}$) as a function of s is what we called the impulse-response function. However, note that the elements of *ε*_*t*_ are contemporaneously correlated; this is, a positive shock in the first variable (say, *ε*_1*t*_>0) is related to the values of *ε*_2*t*_, *ε*_3*t*_,…,*ε*_*nt*_. In other words, in general, Ω is not a diagonal matrix. One evident approach is to decompose the VAR innovations into a set of uncorrelated components (what is known as the orthogonalized impulse-response function). To this end, recall that Ω = V(*ε*_*t*_) is a positive definite symmetric (nxn) matrix, hence, it has a unique representation of the form Ω = *ADA*′; where A is a lower triangular matrix A with 1s in the main diagonal, and D is a diagonal matrix.

Let *u*_*t*_ be a (nx1) vector such that *u*_*t*_ = *A*^−1^*ε*_*t*_. As *ε*_*t*_ is white noise, it is uncorrelated with its own lags. Hence, *u*_*t*_ is also uncorrelated with its own lags and lagged *Y*_*t*_ values. Moreover, notice that $E(u_{t}u_{t}’)=A^{-1}E(\varepsilon_{t}\varepsilon_{t}^{\prime })A^{-1}’=D$; in other words, as D is a diagonal matrix, the elements of *u*_*t*_ are uncorrelated. Also, notice that *Au*_*t*_ = *ε*_*t*_, hence the elements under the main diagonal in A capture the covariances among contemporaneous shocks. Let *a*_*j*_ be the *j*th column of the matrix A, then the sequence of Ψ_s_*a*_*j*_ as a function of s is the orthogonalized impulse-response function.

Finally, consider the Cholesky decomposition of Ω

Ω=PP′ [Eq 6]

Where P = AD^0.5^; P collects the standard deviations of *u*_*t*_ in its main diagonal. Let *p*_*j*_ be the *j*th column of P, then $\Psi_{s}p_{j}=\Psi_{s}a_{j}\sqrt{V(u_{jt})}$. Thus, this last expression measures the dynamic system’s consequences due to an increase in *Y*_*jt*_ of $\sqrt{V(u_{jt})}$ units. Note that the ordering of the VAR´s variables is relevant for the orthogonalization. Our argument in this paper is that the VIX is a forward-looking implied volatility measure that should precede the network asset´s correlation. As our results in next Section suggest, it seems that the VIX tends to anticipate (Granger-cause) future movements in the MSTL; accordingly, the first variable in the left hand side of our system is the VIX.

## 4 Empirical results

Our empirical results have three parts. Firstly, we report the estimation results of 5 core models (See Table 3, Panel A) using in-sample data. Secondly, we evaluate the forecasting performance with our benchmark models (Table 3, Panel B) and calculate the ENCNEW out-of-sample test of Clark and McCracken [33]. Finally, we present the impulse response function (IRF) and forecasting error variance decomposition results of our core models.

### 4.1 In-sample analysis

Tables 4 and 5 report estimates of core models presented in Table 3 panel A. We consider monthly frequencies and use HAC standard errors [33,35]. We show a negative and statistically significant relationship between the lagged implied volatility and the variation of the global and regional MSTL and PMFGL. In other words, an increase of the implied volatility is a preview of a contraction in the stock market networks. Thus, when investors increase their expectations regarding the volatility of the markets (implied volatility), our interpretation indicates that they make investment decisions that tend towards a standard, like a herd behavior effect, causing asset prices to behave similarly—increasing in consequence, the correlation of the stock indices. From this perspective, the main implication of volatility, as a market sentiment manifestation, is noteworthy. As our results point out, an increase in volatility expectations, seen from a behavioral perspective as a fear feeling, generates an increase in the markets’ correlation, limiting the benefits of portfolio diversification.

**Table 4:** Forecast variation in MSTL and PMFGL with volatility indices.

	VMSTL-G					VPMFGL-G				
	1	2	3	4	5	6	7	8	9	10
C	-0.003	-0.004	-0.004	-0.003	-0.004	-0.004	-0.004	-0.004	-0.003	-0.004
	0.009	0.009	0.009	0.009	0.009	0.009	0.009	0.009	0.009	0.009
VVIX(-1)	-0.184***			-0.191*		-0.189***			-0.190**	
	**0.059**			**0.080**		**0.061**			**0.083**	
VVSTOXX(-1)		-0.149*		0.042			-0.152**		0.055	
		**0.071**		0.101			**0.068**		0.099	
VVXJ(-1)			-0.129*	-0.037				-0.145***	-0.060	
			**0.052**	0.062				**0.052**	0.066	
PC2(-1)					-0.023***					-0.024***
					**0.009**					**0.009**
R-squared	0.312	0.292	0.290	0.314	0.306	0.321	0.300	0.303	0.324	0.316
Adjusted R-squared	0.267	0.246	0.243	0.261	0.260	0.276	0.254	0.258	0.272	0.271
S.E. of regression	0.129	0.131	0.131	0.130	0.130	0.133	0.135	0.135	0.134	0.134
Sum squared resid	3.320	3.418	3.429	3.313	3.351	3.546	3.653	3.636	3.530	3.570
Log likelihood	140.957	137.840	137.508	141.176	139.964	133.923	130.759	131.251	134.401	133.223
F-statistic	6.949	6.307	6.240	6.001	6.742	7.223	6.564	6.665	6.284	7.076

In-sample analysis with monthly data and core specification from Table 3. In all models we included, yet not show, an AR(12) that stands for lag monthly of the dependent variable.

*p < 10%

**p < 5%

***p < 1%. Source: Authors’ elaboration.

**Table 5:** Forecast variation in regional MSTL with volatility indices.

	MSTL—AMERICA					MSTL—EUROPE					MSTL—ASIA-OCEANIA				
	1	2	3	4	5	6	7	8	9	10	11	12	13	14	15
C	-0.005	-0.005	-0.005	-0.005	-0.005	-0.005	-0.005	-0.004	-0.004	-0.005	-0.003	-0.003	-0.003	-0.003	-0.003
	0.010	0.011	0.011	0.010	0.011	0.015	0.015	0.015	0.015	0.015	0.009	0.009	0.010	0.009	0.009
VVIX(-)	-0.231***			-0.191*		-0.161*			-0.227*		-0.219***			-0.242**	
	**0.070**			**0.100**		**0.087**			**0.131**		**0.057**			**0.094**	
VVSTOXX(-1)		-0.214**		-0.054			-0.054		0.353**			-0.194***		-0.023	
		**0.077**		0.106			0.093		**0.143**			**0.058**		0.102	
VVXJ(-1)			-0.152**	0.002				-0.212***	-0.279***				-0.110**	0.063	
			**0.063**	0.068				**0.067**	**0.090**				**0.054**	0.072	
PC2(-1)					-0.029***					-0.022*					-0.026***
					**0.009**					**0.011**					**0.008**
R-squared	0.387	0.377	0.361	0.388	0.383	0.315	0.304	0.328	0.345	0.316	0.393	0.374	0.349	0.396	0.379
Adjusted R-squared	0.347	0.336	0.319	0.342	0.343	0.271	0.259	0.284	0.295	0.271	0.354	0.334	0.306	0.350	0.338
S.E. of regression	0.156	0.157	0.159	0.156	0.156	0.219	0.220	0.217	0.215	0.219	0.135	0.137	0.140	0.135	0.136
Sum squared resid	4.820	4.905	5.030	4.814	4.856	9.518	9.675	9.346	9.102	9.512	3.608	3.721	3.875	3.593	3.696
Log likelihood	101.233	99.388	96.694	101.373	100.460	28.781	27.036	30.726	33.538	28.842	132.086	128.795	124.493	132.536	129.521
F-statistic	9.676	9.247	8.634	8.330	9.496	7.049	6.685	7.461	6.924	7.062	9.928	9.160	8.192	8.610	9.328

In-sample analysis with monthly data and core specification from Table 3. In all models we included, yet not show, an AR(12) that stands for lag monthly of the dependent variable.

*p < 10%

**p < 5%

***p < 1%. Source: Authors’ elaboration.

To avoid the loss of information, we measure the behavior of the global network of assets using two measurements. Table 4, panel A represents the network with the MSTL, while panel B, shows the network with the PMFGL. Using both measures has the advantage that the MSTL only includes the most significant correlations in the network, while the PMFG includes all the correlations. As shown, the results do not vary much between the two measures. In the case of the MSTL, the three implicit volatility indices are negative and statistically significant (See Table 4 column 1–3), although the VIX (beta: -0.184, se: 0.059) presents greater magnitude and statistical significance compared to the others volatility indices. The estimates of the PMFG provide similar results (See Table 4 column 6–8) where the VIX maintains its preponderance in magnitude and statistical significance (beta: -0.189, se: 0.061), but the VSTOXX (beta: -0.152, se: 0.059) and the VXJ (beta: -0.145, se: 0.052) increase their statistical significance.

We estimate a core model including the three volatility indices (See Table 4, columns 4 and 9) to discriminate between the three volatility indices. The results are consistent in positioning the VIX as the volatility index with the highest predictive power and statistical significance (beta: -0.190, se: 0.009). Additionally, in terms of the adjusted coefficient of determination, the improvement is marginal when comparing the estimation models with the VIX and the estimation models with the three volatility indicators, providing additional arguments of the VIX’s relevance to the other volatility indicators.

To further explore the combined effect of the three volatility indicators we organized a final estimate. Previously, we performed a principal components method to extract the information from the three volatility indicators. Table 4 columns 5 and 10 show a negative and significant coefficient (beta: -0.023, se: 0.009) for the MSTL and (beta: -0.024, se: 0.009) for the PMFGL. Although both models do not contribute additional information when comparing the adjusted coefficients of detection with models that only incorporate the VIX.

Finally, we analyze the above models considering regional stock markets. As Table 5 shows, results are similar according to the geographical areas. The VIX remains the predominant index to predict changes in America’s correlation network (beta: -0.231, se: 0.070), Asia, and Oceania (beta: -0.219, se: 0.057). For Europe’s case, the results are not consistent with what was expected since the VSTOXX index does not present statistical significance to produce changes in Europe’s network, compared to its Japanese counterpart, the VXJ (Beta: -0.212, se: 0.067). Despite these differences, our evidence indicates that the VIX and the main factor constructed between the three volatility indices provide the possibility for predicting changes in the network of correlations of both the global stock assets and each region.

### 4.2 Out of sample

Tables 6 and 7 exhibit the ENCNEW test results in out-of-sample exercise for the Americas, Europe, and Asia-Oceania. To add more rigor to the test, we separated the American zone into two sub-zones, North America and Latin-America. These tables focus on the benchmark models described in Table 3, panel B. The results correspond to the statistical difference between the benchmark model presented in Table 3 panel B (with VMSTL and VPMFGL) versus the core models presented in Table 2 panel A, when the number of observations to make the forecast 40% (*P/R = 0*.*4*), 50% (*P/R = 1*), and 67% percent (*P/R = 2*) of the total sample.

**Table 6:** Forecast variation in MSTL with volatilities indexes.

Panel (A) VVIX model							
(1)	(2)	(3)	(4)	(5)	(6)	(7)	(8)
P/R	GLOBAL	ASIAOC	EUROPE	LATAM	NORTH AMERICA	AMERICA	GLOBAL*
0.4	2.37**	2.81**	1.34*	1.92**	3.37***	2.47**	2.40**
1	8.49***	14.75***	2.01**	7.47***	10.15***	9.45***	8.67***
2	2.22**	2.47**	0.46	1.90**	2.67**	2.61**	2.20**
Panel (B) VSTOXX model							
P/R	GLOBAL	ASIAOC	EUROPE	LATAM	NORTH AMERICA	AMERICA	GLOBAL*
0.4	1.26*	2.07**	-0.71	1.33*	2.00**	1.96**	1.23*
1	3.44***	8.06***	-0.48	4.72***	5.71***	6.77***	3.46***
2	1.03*	1.82***	-1.96	1.29*	1.04*	1.65**	0.93
Panel (C) VXJ model							
P/R	GLOBAL	ASIAOC	EUROPE	LATAM	NORTH AMERICA	AMERICA	GLOBAL*
0.4	1.77**	1.33*	2.35**	0.82	2.18**	1.74**	2.04**
1	4.98***	2.99**	6.40***	2.27**	11.36***	4.99***	6.30***
2	1.20*	0.92	1.29*	0.81	1.27*	1.44*	1.44*

Out-of-sample analysis with monthly data, (P/R = 0.4). 10%, 5%, and 1% critical values are 0.685, 1.079, and 2.098, respectively, when there is only one excess parameter. (P/R = 1). 10%, 5%, and 1% critical values are 0.984, 1.584, and 3.209, respectively, when there is only one excess parameter. (P/R = 2). 10%, 5%, and 1% critical values are 1.280, 2.085, and 4.134, respectively, when there is only one excess parameter. P represents the number of one-step-ahead forecasts, R the sample size of the first estimation window. All models are evaluated with AR(12), benchmark corresponds to model 1.

*p < 10%

**p < 5%

***p < 1%. Source: Authors’ elaboration.

**Table 7:** Forecast variation in MSTL with volatilities principal component.

Panel (A) Principal component model							
(1)	(2)	(3)	(4)	(5)	(6)	(7)	(8)
P/R	GLOBAL	ASIAOC	EUROPE	LATAM	NORTH AMERICA	AMERICA	GLOBAL*
0.4	1.90**	2.29**	1.39*	1.49*	2.79**	2.23**	2.05**
1	7.36***	10.13***	2.35**	6.34***	10.99***	9.18***	7.90*
2	1.59**	1.95**	0.31	1.44*	1.95**	1.99**	1.65**
Panel (B) VVIX–VSTOXX—VXJ model							
P/R	GLOBAL	ASIAOC	EUROPE	LATAM	NORTH AMERICA	AMERICA	GLOBAL*
0.4	1.84	2.36*	2.74**	1.67	1.86	2.03*	1.93*
1	6.39***	10.36***	5.52***	6.57***	6.01***	7.55***	6.55***
2	1.98**	2.63**	3.54***	1.12	2.53**	2.15**	2.28**

Forecasting VMSTL and PMFGL changes with volatilities indexes. Panel A show out-of-sample analysis with monthly data, (P/R = 0.4). 10%, 5%, and 1% critical values are 0.685, 1.079, and 2.098, respectively, when there is only one excess parameter. (P/R = 1). 10%, 5%, and 1% critical values are 0.984, 1.584, and 3.209, respectively, when there is only one excess parameter. (P/R = 2). 10%, 5%, and 1% critical values are 1.280, 2.085, and 4.134, respectively, when there is only one excess parameter. Panel B show out-of-sample analysis with monthly data, (P/R = 0.4). 10%, 5%, and 1% critical values are 1.285, 1.865, and 3.098, respectively, when there are three excess parameters. (P/R = 1). 10%, 5%, and 1% critical values are 1.905, 2.709, and 4,574, respectively, when there are three excess parameters. (P/R = 2). 10%, 5%, and 1% critical values are 2.366, 3.564, and 5.805, respectively, when there are three excess parameters. P represents the number of one-step-ahead forecasts, R the sample size of the first estimation window. All models are evaluated with AR(12), benchmark corresponds to model 1.

*p < 10%

**p < 5%

***p < 1%. Source: Authors’ elaboration.

Table 6, panel A, shows the contrast tests between the benchmark model (Table 3, Panel B) and the first core model (Table 3, Panel A, row 1). For the case of predicting the changes in the global asset network measured by the MSTL (Table 6, panel A, column 2) and by the PMFGL (Table 6, panel A, column 8) with the lagged one-period variation of the VIX. We reject the null hypothesis, which means that the forecast model that incorporates the lag of the VIX variation is statistically better than the benchmarks models. This result is repeated for all regions, although with a significance level that fluctuates between 1% and 10%.

Regarding VSTOXX analysis (See Table 3, Panel A, row 2), the core models (incorporating the one-month lag variation of the VSTOXX) obtain worse results than the VIX. Like the tests within the sample, we observe an inconsistency with our expectations for Europe since the models that incorporate the VSTOXX have worse performance than the benchmark. Notwithstanding this, at the global level, as in the rest of the regions, the VSTOXX models present an acceptable statistical significance at *P/R = 0*.*4* that fluctuates between 5% and 10%, demonstrating a moderate-acceptable predictive power.

Similarly, we find results regarding the predictive power of the VJX (See Table 3, Panel A, row 3). The predictive model that includes the VJX lag only for the Latin-America region turns out to be statistically significant only in *P/R = 1*. The VJX shows a statistically significant predictive power that fluctuates between 1% and 10% for the rest of the global and regional samples.

Finally, we assess the predictive power of models that consider the combined effect of the volatility indices (See Table 3, row 4–5). As shown, we observe favorable results for the principal component model compared to the model that includes all the indices separately. Table 7 shows that for the principal component (see Table 7, panel A), the models are statistically superior to the benchmark model for the global and regional samples. In contrast, models with the three volatility indices separately (See Table 7, panel B) are only significant for Europe, Asia-Oceania, America, and globally when measured by the PMFGL. The significance fluctuates between 5% and 10%.

### 4.3 VAR

Table 8 exhibits our results for a VAR(2) using the VIX and the networks measures (MSTL) of each region. We select the order of the VAR (p = 2) using the Hannan-Quinn Information criteria. Our focus here is to study the Granger-causality relationships; in this atheoretical VAR, we may find Granger-causality in one direction (say, the VIX predicting the MSTL) in the opposite direction (the MSTL predicting the VIX), or both. We emphasize that we are not attempting to identify the channels of transmissions by any means; on the contrary, we are just interested in studying the dynamic effects of the system (e.g., establishing if one variable helps forecast the other beyond a simple autoregressive benchmark).

**Table 8:** VAR Results for VIX and MSTLs models.

*(1)*	*(2)*	*(3)*	*(4)*	*(5)*	*(6)*	*(7)*	*(8)*
	VIX	MSTL	MSTL AME	MSTL ASIOC	MSTL EUR	MSTL LAT	MSTL NAM
VIX(-1)	-0.213***	-0.161***	-0.189***	-0.202***	-0.095	-0.190***	-0.284***
	(0.083)	(0.055)	(0.065)	(0.059)	(0.094)	(0.066)	(0.098)
VIX(-2)	-0.100	-0.037	-0.011	-0.053	-0.036	-0.007	-0.067
	(0.084)	(0.056)	(0.065)	(0.059)	(0.095)	(0.067)	(0.099)
MSTL(-1)	0.581	-1.296***	-0.958*	-0.505	-0.580	-0.758	-0.726
	(0.714)	(0.473)	(0.557)	(0.504)	(0.806)	(0.569)	(0.840)
MSTL(-2)	0.699	-1.090**	-1.008*	-0.785	-0.950	-0.461	-1.693**
	(0.718)	(0.476)	(0.560)	(0.507)	(0.811)	(0.572)	(0.845)
MSTL AME(-1)	-0.433	0.339	-0.075	0.300	0.100	0.625*	-0.360
	(0.447)	(0.296)	(0.349)	(0.316)	(0.505)	(0.356)	(0.526)
MSTL AME(-2)	-0.311	0.098	0.008	0.105	-0.046	0.051	0.268
	(0.452)	(0.299)	(0.352)	(0.319)	(0.510)	(0.360)	(0.531)
MSTL ASIOC(-1)	-0.048	0.325	0.526**	-0.405*	0.242	0.390	0.417
	(0.302)	(0.200)	(0.235)	(0.213)	(0.341)	(0.240)	(0.355)
MSTL ASIOC(-2)	-0.142	0.356*	0.491**	-0.046	0.555	0.231	0.898**
	(0.307)	(0.203)	(0.239)	(0.216)	(0.346)	(0.244)	(0.361)
MSTL EUR(-1)	-0.172	0.216*	0.242*	0.179	-0.360*	0.181	0.220
	(0.182)	(0.121)	(0.142)	(0.128)	(0.206)	(0.145)	(0.214)
MSTL EUR(-2)	-0.250	0.234**	0.262*	0.262**	-0.037	0.146	0.434**
	(0.181)	(0.120)	(0.141)	(0.128)	(0.205)	(0.144)	(0.213)
MSTL LAT(-1)	-0.142	-0.010	-0.164	-0.021	0.276	-0.840***	0.443
	(0.294)	(0.195)	(0.230)	(0.208)	(0.332)	(0.235)	(0.346)
MSTL LAT(-2)	-0.066	0.149	0.043	0.143	0.343	-0.210	0.205
	(0.293)	(0.194)	(0.229)	(0.207)	(0.331)	(0.234)	(0.345)
MSTL NAM(-1)	0.166	-0.131*	-0.194**	-0.131*	-0.108	-0.201**	-0.678***
	(0.104)	(0.069)	(0.081)	(0.074)	(0.118)	(0.083)	(0.123)
MSTL NAM(-2)	0.087	-0.022	-0.065	-0.034	-0.004	0.004	-0.393***
	(0.104)	(0.069)	(0.081)	(0.074)	(0.118)	(0.083)	(0.123)
Constant	0.001	-0.001	-0.002	-0.001	-0.002	-0.001	-0.004
	(0.014)	(0.009)	(0.011)	(0.010)	(0.015)	(0.011)	(0.016)
F-statistic	1.615	4.407	7.412	6.028	4.121	6.144	10.058
Adj. R-squared	0.037	0.177	0.288	0.241	0.164	0.245	0.364

VAR analysis with monthly data and core specification from Table 3. Akin to this exercise, in unreported results, we also consider VAR using the VSTOXX and the VXJ instead of the VIX. Our results are very similar and they are available upon request.

*p < 10%

**p < 5%

***p < 1%. Source: Authors’ elaboration.

Some highlights of Table 8 are worth mentioning. First, the relationship between the MSTL in each region and the first lag of the VIX is negative in all VAR equations. These results are consistent with our main argument in this paper: the VIX is a forward-looking measure of implied volatility that precedes a higher correlation among the network’ assets. Second, the first lag of the VIX is significant at the one percent level in seven out of eight cases: this is consistent with the idea that the VIX may Granger-cause the network’s correlations. Third, none of the lagged MSTLs network measures is significant in Table 8 Column 2; in other words, we do not find evidence that the MSTL Granger-cause the VIX. Finally, we notice differences between the VIX equation (Table 8 Column 2) and MSTLs equations (Table 8 Columns 3–8) in terms of the adjusted: for Table 8 Columns 3–8, the adjusted goes from 0.164 to 0.364, while the adjusted in Column 2 is only 0.037. All in all, the results of Table 8 suggest the existence of Granger-causality, and this predictability seems to go from the VIX to the network asset’s correlations.

**Fig 3:**
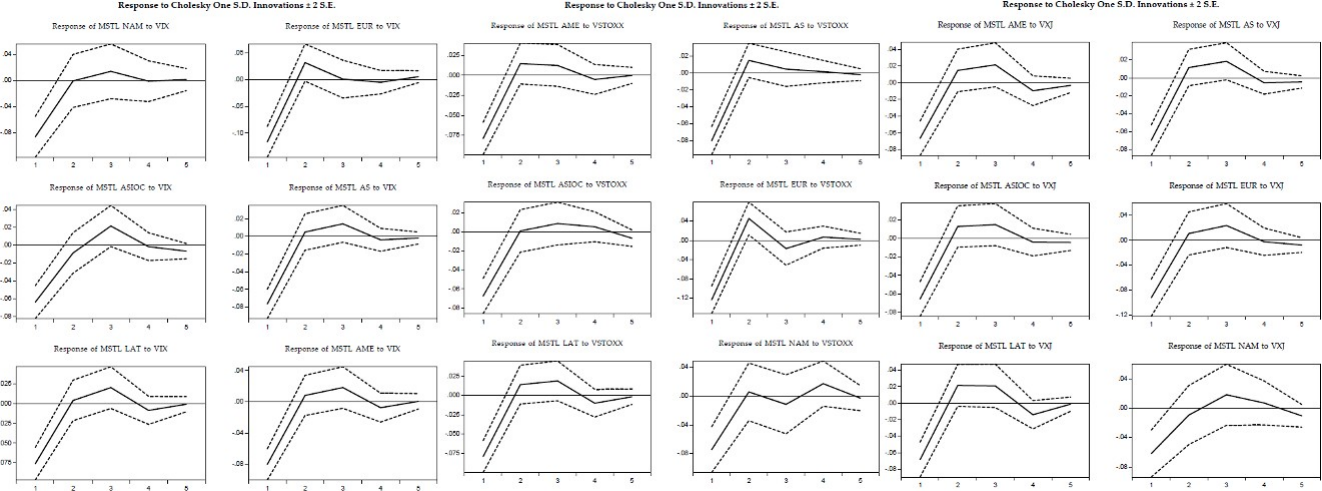
Impulse response graphs. Note: This figure depicts the impulse response exercise from a shock in VIX over the MSTLs of North-America (NAM), Europe (EUR), Asia-Oceania (ASIOC), All-shares (AS), Latin America (LAT), and America (AME, North and Latin America). This figure depicts the impulse response exercise from a shock in VSTOXX over the MSTLs of America (AME, North and Latin America), All-shares (AS), Asia-Oceania (ASIOC), Europe (EUR), Latin America (LAT), and North America (NAM). This figure depicts the impulse response exercise from a shock in VXJ over the MSTLs of America (AME, North and Latin America), All-shares (AS), Asia-Oceania (ASIOC), Europe (EUR), Latin America (LAT), and North America (North America).

Fig 3 shows the impulse-response function derived from our VAR. We exhibit the response of the MSTL of each region after a shock of one standard deviation in the VIX. Consistent with our previous findings, the MSTL in each region responds negatively (i.e., they tend to be more correlated since the length of the MST shrinks) after a positive shock in the VIX. Moreover, in all cases, this response is significant one period after the shock. Notably, in each region, the shock is rapidly absorbed after the first period. In other words, there are no significant differences with the counter-factual two months after the shock.

Finally, following [19] Table 9 considers how each of the orthogonalized disturbances contributes to the mean squared error (MSE) in the h-periods-ahead forecasts. First, we notice that most of the MSTLs tend to be very autoregressive; in each MSTL, their lags explain most of the variance in the MSE. For instance, about sixty percent of the variance in the MSTL of Europe is explained by the same network; an even more extreme case is the MSTL of North America, in which their lags are accounting for about ninety percent of the MSE variance. Second, notice that the VIX explains an essential proportion of the variance in most cases: for h = 1, it ranges from 12.89 through 31.89 percent across all exercises. Finally, with only two exceptions, the VIX is always the second most important source of variance for the MSTLs.

**Table 9:** Forecast error variance decomposition results for the period from 2001 to 2020.

*(1)*	*(2)*	*(3)*	*(4)*	*(5)*	*(6)*	*(7)*	*(8)*
h	VIX	MSTL	MSTL AME	MSTL ASIOC	MSTL EUR	MSTL LAT	MSTL NAM
Variance Decomposition of MSTL NAM							
1	12.894	0.000	0.000	0.000	0.000	0.000	87.106
2	8.499	0.194	0.139	0.281	0.146	0.388	90.352
3	8.463	0.337	0.873	0.273	0.168	0.470	89.416
4	8.213	0.349	1.229	0.748	0.264	1.218	87.978
Variance Decomposition MSTL EUR							
1	25.525	0.000	0.000	0.000	64.319	0.000	10.157
2	22.494	0.344	0.015	0.094	63.718	0.858	12.477
3	22.307	0.717	0.017	0.405	63.186	0.854	12.515
4	21.797	0.847	0.029	1.417	62.316	1.225	12.369
Variance Decomposition of MSTL ASIOC							
1	19.493	0.000	0.000	64.529	6.879	0.000	9.100
2	15.179	0.068	0.311	65.924	5.478	0.344	12.695
3	16.471	0.111	0.322	64.526	5.368	0.556	12.646
4	15.938	0.192	0.360	64.278	5.249	1.281	12.701
Variance Decomposition of MSTL							
1	31.886	8.017	0.000	14.525	24.837	0.000	20.734
2	25.880	8.505	0.480	13.961	22.870	0.634	27.671
3	26.319	8.364	0.549	13.735	22.478	0.707	27.848
4	25.984	8.619	0.561	13.567	22.174	1.357	27.737
Variance Decomposition of MSTL LAT							
1	21.752	40.287	0.000	2.855	4.167	19.556	11.383
2	16.527	41.485	1.055	2.211	3.562	18.749	16.411
3	16.944	39.883	1.625	2.292	3.484	18.164	17.608
4	16.974	40.116	1.635	2.271	3.474	18.017	17.513
Variance Decomposition of MSTL AME							
1	25.311	29.274	3.719	1.685	3.608	5.504	30.899
2	18.350	31.225	2.685	1.294	2.755	4.554	39.138
3	18.479	30.605	2.590	1.551	2.665	4.713	39.397
4	18.474	30.649	2.575	1.565	2.666	4.914	39.157

This table reports the results of forecast error variance decomposition (percentage points) among VIX, MSTL, and MSTL of each region. The variance decomposition is based on the Cholesky ordering VIX, MSTL, and each regional MSTL. Source: Authors’ elaboration.

In summary, we think the message of this section is clear and consistent with our forecasting exercises: a) the VAR, impulse-response functions, and MSE variances decomposition suggest Granger-causality from the VIX to the MSTLs (but not in the opposite direction), b) this relationship is negative (more volatility precedes a more correlated network), and c) the predictive content of the VIX goes beyond purely autoregressive benchmarks.

## 5 Conclusions

Indices based on implied market volatility expectations, such as the VIX, have been extensively studied by academics and used by practitioners. Among their main attributes, these indices are essential to measuring the degree of near-term uncertainty of the markets, possessing the ability to predict the volatility of financial assets’ returns, and providing useful information to market participants and regulators.

Notwithstanding, the indices’ predictive power for the synchronization of the financial markets is still unknown to our best understanding. Although the literature shows links between implied market volatility, stock returns, and economic uncertainty, there is still a necessity for shedding light regarding the predictive power of the volatility indices. As the latest financial turmoil episodes show, we are not fully aware of the factors behind periods of high synchronization of returns.

In this research, we explore the predictive power of the three main implicit volatility indices of the world, both separately and together, to study their impact on the stock network made up of the correlations of returns for the most relevant world equity indices. These networks serve as a vehicle to the quantitative dynamic representation of the broad phenomenon of synchronization of financial markets.

Our main results indicate a strong predictive power of the implicit volatility indicators on the synchronization of stocks’ returns. Being the VIX, the index that exhibits superior predictive performance compared to VSTOXX and VXJ alternatives. The latter occurs in both the regional and global networks. We observe that an increase in the market’s implied volatility is a predictor of an increase in the synchronization of the stock markets in the following month.

To study the existence of Granger-causality from the VIX, VSTOXX, and VXJ to the MSTL, we apply a Structural VAR, finding strong evidence that the implicit volatility indices generate stock market synchronization. We conjecture that an increase in the implied volatility is a sign of rising uncertainty and future greater volatility and financial risk expectations. As the literature shows, this factor moves investors to make similar financial decisions. This behavior, as a herding factor, causes prices of financial assets to synchronize.

From an investor perspective, an increase in synchronization reduces the chances of well-diversifying investment portfolios, increasing the cost of managing risks and reducing the long-term return on investments. From a financial regulator’s point of view, the synchronization of financial markets is important because an increase in it would have dangerous consequences on the risk of financial contagion in markets. Our work helps them in the task of monitoring this phenomenon dynamically. Finally, regulators, financial institutions, and investors, in general, should measure, monitor, and estimate synchronization to improve decision-making and take actions in advance for diminishing the impact of shocks.

A natural extension of this work relates to the development of structural financial and economic models that help explain the factors behind the phenomenon of synchronization of returns. Although our study shows a Granger causality phenomenon interpreted as the predictive power of volatility indices on the stocks’ returns synchronization, this is the first step for gaining comprehensive knowledge above this peculiar financial market behavior.

Another extension is to study the link between Quantitative easing (QE) and stock market synchronization. The Federal Reserve Bank’s actions during turmoil periods, such as the 2008–09 crisis or covid pandemic 2020–21, executing quantitative easing (QE) policy, have had a significant impact on the behavior of worldwide financial markets in terms of returns and volatility ([19]). Analyzing volatility spillover networks, these authors find that the US markets are a powerful spillover source towards the rest of financial markets that under certain conditions could destabilize markets, enhancing global systemic risk. Specifically, through Treasury Bonds rates, QE provokes that TBond volatility acts as an exogenous source of spillover volatility in contemporaneous time, influencing VIX. They also state that US stock volatility (VIX) is a prime source of volatility towards other stock markets. Considering those above, we conjecture that QE would probably impact the stock network’s behavior in two ways in our research framework. Firstly, directly affecting the synchronization of the markets, as Yang and Shou (2016) evidence. Secondly, indirectly influencing the VIX, which will impact the rest of the other markets’ synchronization, as our results indicate.
